# The Effect of Protein Supplementation and Playing Time on Recovery Kinetics During a Congested Basketball Schedule

**DOI:** 10.3390/nu17010128

**Published:** 2024-12-31

**Authors:** Dimitrios Pantazis, Alexandra Avloniti, Draganidis Dimitrios, Theodoros Stampoulis, Maria Protopapa, Christos Kokkotis, Dimitrios Balampanos, Sotirios Arsenis, Athanasios Poulios, Konstantinos Papanikolaou, Vassiliki C. Laschou, Panagiotis Tsimeas, Georgios Vitkas, Nikolaos Papaspanos, Nikolaos Zaras, Asimenia Gioftsidou, Paraskevi Malliou, Maria Michalopoulou, Athanasios Z. Jamurtas, Ioannis G. Fatouros, Chatzinikolaou Athanasios

**Affiliations:** 1 Department of Physical Education and Sport Science, School of Physical Education, Sport Science and Occupational Therapy, Democritus University of Thrace, 69100 Komotini, Greece; dpantazi@phyed.duth.gr (D.P.); alavloni@phyed.duth.gr (A.A.); tstampou@phyed.duth.gr (T.S.); mprotopa@phyed.duth.gr (M.P.); ckokkoti@affil.duth.gr (C.K.); dimibala10@phyed.duth.gr (D.B.); sarsenis@gmail.com (S.A.); geovitkas@gmail.com (G.V.); nmpapaspanos@yahoo.gr (N.P.); nzaras@phyed.duth.gr (N.Z.); agioftsi@phyed.duth.gr (A.G.); pmalliou@phyed.duth.gr (P.M.); michal@phyed.duth.gr (M.M.); 2 Department of Physical Education and Sport Science, School of Physical Education, Sport Science and Dietetics, University of Thessaly, 43100 Trikala, Greece; ddraganidis@pe.uth.gr (D.D.); athanpoul@gmail.com (A.P.); kpapanikolaou@uth.gr (K.P.); lavassia123@gmail.com (V.C.L.); ptsimeas@pe.uth.gr (P.T.); ajamurt@pe.uth.gr (A.Z.J.); ifatouros@pe.uth.gr (I.G.F.); 3 Department of Life Sciences, School of Life and Health Sciences, University of Nicosia, Nicosia 1700, Cyprus

**Keywords:** basketball, congested schedule, protein supplementation, recovery, muscle function, glutathione

## Abstract

Background/Objectives: Despite being widely promoted, protein supplementation’s overall effectiveness during demanding basketball schedules remains unclear. This study investigated whether increased protein intake can accelerate recovery of muscle function during a 6-day congested basketball microcycle consisting of three consecutive games while accounting for the impact of playing time. Methods: In a randomized, two-trial, cross-over, double-blind repeated measures design, eighteen male basketball players were assigned to a high (High PT) or a moderate (Mod PT) playing time group and participated in two trials, receiving daily either milk protein (PRO trial) or an isoenergetic amount of carbohydrates. Each trial included three consecutive games (days 1–3) and a 72 h recovery period following Game 3 (days 4–6), during which players participated in low-load practice sessions. Isometric and isokinetic peak torque of knee extensors and flexors in the dominant limb, serum creatine kinase (CK) concentration, and erythrocyte glutathione (GSH) levels were assessed prior to each game and practice session. Results: CK increased (*p* < *0.01*) on game days in both groups but recovered earlier in Mod PT compared to High PT. Both eccentric and concentric peak torque was impaired (*p* < *0.01*) up to 24–48 h post-G3 in a velocity-dependent manner. Eccentric peak torque of knee flexors at 60°/s declined to a greater extent in High PT compared to Mod PT (*p* < *0.01*). Protein supplementation resulted in higher erythrocyte GSH concentration at pre-G2 (*p* < *0.05*) and pre-G3 (*p* < *0.05*) compared to placebo in both groups but did not affect any of the study outcomes. Conclusions: Increased protein intake during a congested basketball schedule increases erythrocyte GSH concentration but does not accelerate recovery of muscle function.

## 1. Introduction

Basketball is an intermittent sport characterized by continuous activity changes, where high-intensity multidirectional actions such as changes of direction, rapid accelerations and decelerations, sprints, and jumps are alternated with periods of lower intensity, like jogging, walking, or standing [1,2,3]. The powerful actions include a considerable number of eccentric muscle contractions that have been linked to exercise-induced muscle trauma (EIMT) [4,5]. The previous work by Chatzinikolaou et al. [6] revealed that a single basketball game induces EIMT and inflammatory response accompanied by performance deterioration lasting ~24–48 h. Of note, these findings were reinforced further by subsequent evidence showing elevated serum EIMT indices and reduction in performance for 37–48 h after a basketball game [7,8,9].

In modern basketball, though, the physiological demands have risen dramatically as players participate in a greater number of official games with a higher frequency, increasing the number of weekly microcycles consisting of 2 to 4 games during the competitive period [1,10]. For instance, such a congested schedule, including either two games on two consecutive days or multiple games (up to 5 games) on consecutive days, is encountered in the National Basketball Association (NBA) or special types of tournaments, such as cups or qualifiers, lower-division leagues, national cup competitions, and various U18 tournaments [11,12]. Therefore, it is evident that during several weeks within a season, players are repeatedly trained and compete with less than 24–48 h of recovery, which is insufficient to allow restoration of muscle homeostasis following game-induced EIMT [6,7,8,9]. Participation in multiple games with inadequate recovery during a congested basketball schedule increases remarkably the workload and is associated with a higher risk of injury in basketball players [11,13,14,15]. However, it is the minutes played in each game rather than the number of games played per se that affect the workload imposed on players and, consequently, the injury risk during a congested basketball calendar [16]. Indeed, Orringer and Pandya [15] observed that increasing the minutes played during consecutive games increases the injury risk. So far, the impact of a congested basketball schedule on EIMT and performance indices has been examined during a 3-day tournament and a subsequent 24 h recovery period [17,18]. Muscle soreness (DOMS) and plasma concentrations of creatine kinase, interleukin-6, and interleukin-10 were markedly elevated, while agility, speed, and flexibility declined up to 24 h post-game 3 [17,18], indicating that the muscle healing process is incomplete, and performance is not recovered 24 h after a format of 3 games in 3 consecutive days [17,18].

Several recovery strategies, including load management (expressed as minutes played per game), compression, cold-water immersion, physiotherapy methods, and nutritional interventions, have been examined in terms of their efficacy in accelerating skeletal muscle and performance recovery during a congested team sports schedule [14,17,18,19,20]. Nutritional strategies, particularly those focusing on increased carbohydrate, fluid, and protein intake, have been shown to support the recovery process of muscle function and physical performance in team sport athletes [21]. Particular attention has been paid to protein, given its ability to maximize muscle protein synthesis and promote a positive net protein balance during recovery from exercise [22,23]. In addition, most high-quality protein supplements are rich in sulfur-containing amino acids (glycine, glutamate, cysteine), which are precursors for the synthesis of glutathione (GSH) [24], a pivotal antioxidant that regulates cellular redox homeostasis and mitigates the inflammatory and oxidative stress response to EIMT [25,26]. Increased GSH availability through N-acetylcysteine supplementation has been shown to attenuate the inflammatory response and performance impairment during the short-term (24–72 h) recovery period after eccentric exercise-induced muscle trauma [25,26]. Interestingly, previous research in soccer has shown that increased daily protein intake during a congested microcycle can accelerate the recovery of soccer-specific performance by eliciting an antioxidant effect [27]. The efficacy of protein intake in enhancing skeletal muscle and performance recovery in basketball players remains obscure. To the best of our knowledge, only one study has examined the effectiveness of a pre-game (simulated basketball game) protein–carbohydrate co-ingestion in alleviating muscle damage and performance deterioration over a subsequent 24 h recovery period in basketball players [28].

Therefore, the aim of the present study was to investigate whether increased protein intake can mitigate symptoms of EIMT and performance recovery kinetics during a 6-day congested basketball microcycle consisting of three games in three consecutive days. Secondly, this study aimed to account for the impact of playing time during each game on EIMT and performance fluctuations.

## 2. Materials and Methods

### 2.1. Experimental Design

**Figure 1:**
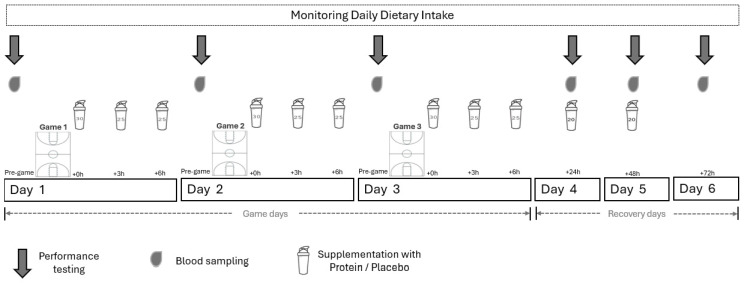
Experimental flowchart. The athletes were divided into two trials (Trial A: Protein and Trial B: Placebo), each consisting of two groups based on playing time (High and Moderate). After a 15-day washout period, the athletes were assigned to the alternate trial.

Figure 1 illustrates the experimental flowchart of this study. A randomized two-trial, cross-over, double-blind repeated measures design was applied. The study was conducted 14 days after the completion of the in-season basketball period. Prior to each trial, participants underwent an assessment of their anthropometrics (body height and body mass), body composition, resting metabolic rate, physical performance (including Yo-Yo IE and IR, countermovement jump, 5 m and 10 m speed, *t*-Test and bench press, and back squat 1RM testing) and daily dietary intake. Subsequently, a 6-day adaptive period was applied during which players were provided with individualized nutritional plans (based on dietary analysis) to ensure adequate energy (kcal) and macronutrient intake, and participated in low-load basketball practices implementing game tactics (using game styles designed for the upcoming games) and shooting drills. In addition, during the adaptive period, participants were familiarized with the isokinetic dynamometry testing procedures.

Before the experimental trials, participants were randomly assigned to two teams, ensuring equal representation of all basketball playing positions, and competed against each other in every game during the trials. Furthermore, in each team, members were further divided into two subgroups, reflecting moderate (Mod PT) and high (High PT) playing time, according to their position. Each trial comprised a 6-day basketball microcycle consisting of three consecutive matches performed during the first three days, followed by three days of low-load recovery practice sessions. All games included a typical basketball warm-up and four 10 min periods, in adherence with the official rules and regulations adopted by FIBA [29], while practice sessions included basketball tactics and shooting drills in an attempt to reflect the strategies adopted by professional basketball teams during congested schedules. Players were provided daily with protein (PRO trial) or isoenergetic placebo (PLA trial) supplements in both trials. A 15-day washout period was allowed between trials, during which participants were engaged in structured low-load practice sessions guided by the researchers while abstaining from the consumption of any type of sports supplement or ergogenic aid.

In each trial, prior to each game and daily during the 72 h recovery period post-Game 3, participants underwent an assessment of their muscle function using isokinetic dynamometry and provided a resting blood sample for the determination of serum creatine kinase (CK) concentration and erythrocyte glutathione (GSH) levels. Blood samples were collected between 07:00 and 8:00 a.m. in a fasted state. Subsequently, participants consumed a standardized breakfast, and after 2 h of resting, they underwent an assessment of lower-limb muscle function (between 09:00 and 10:00 a.m.). All games and practice sessions took place on the same court, between 1:00 and 3:00 p.m., under controlled conditions (20–23 °C, ~60% humidity), while participants were allowed to drink only water ad libitum.

### 2.2. Participants

A preliminary power analysis (effect size f = 0.50, α error probability of 0.05, power of 0.95) indicated that a sample size of 16 subjects is necessary to detect statistically meaningful treatment effects among repeated measurements in response to participation in basketball games and protein/placebo supplementation. Therefore, twenty-four semi-professional male basketball players were initially approached and interviewed, and eighteen were finally included in the study. Participants’ characteristics at the baseline of each trial are shown in Table 1. To be eligible for the study, volunteers had to meet the following criteria: (1) competed in the top three Greek Basketball divisions over the last ≥4 years; (2) free of musculoskeletal injuries that could affect their performance, illnesses, or metabolic disorders; (3) have not used supplements or medications in the six months preceding the study; (4) be non-smokers; and (5) regularly participate in five to six basketball practice sessions, two full-body weightlifting training sessions, and at least one game per week. Each participant was fully informed about the benefits and potential risks and discomforts associated with the study and provided a signed consent form in advance of participation. All procedures were in accordance with the 1975 Declaration of Helsinki, as revised in 2000, and approval was obtained from the Ethics Committee of the Department of Physical Education and Sport Science, University of Thessaly (1324-7/2/2018).

**Table 1:** Participants’ characteristics.

	PRO—High PT (*n* = 9)	PRO—Mod PT (*n* = 9)	PLA—High PT (*n* = 9)	PLA—Mod PT (*n* = 9)	*p* Value
Playing Time (min)	29:22 ± 3:06	15:21 ± 2:33 ^a^	29:23 ± 3:07	15:22 ± 2:08 ^c^	*0.333*
Height (m)	1.86 ± 0.07	1.90 ± 0.05	1.86 ± 0.07	1.90 ± 0.05	*-*
Body Mass (kg)	85.76 ± 9.16	89.71 ± 9.94	85.68 ± 9.30	89.99 ± 9.76	*0.106*
RMR (kcal/day)	1730.44 ± 287.47	1815.56 ± 154.20	1759.33 ± 327.42	1817.56 ± 153.30	*0.399*
Lean Mass (kg)	64.18 ± 5.6	66.58 ± 4.23	64.14 ± 5.50	66.78 ± 4.41	*0.357*
Fat Mass (kg)	17.07 ± 5.60	17.35 ± 5.55	17.12 ± 5.30	17.01 ± 4.99	*0.418*
Body Mass Index (BMI) (kg/m^2^)	24.67 ± 2.05	24.84 ± 1.81	24.65 ± 2.05	24.92 ± 1.75	*0.97*
YoYo IE2 (m)	1448.89 ± 688.12	1442.22 ± 321.63	1453.33 ± 697.14	1428.89 ± 309.37	*0.421*
YoYo IR2 (m)	613.33 ± 203.96	642.22 ± 167.46	604.44 ± 205.37	655.56 ± 142.05	*0.291*
Countermovement Jump (CMJ) (cm)	38.00 ± 7.42	38.14 ± 5.00	38.33 ± 7.02	38.76 ± 4.61	*0.633*
Speed 5 m (s)	1.025 ± 0.045	1.007 ± 0.061	1.019 ± 0.046	1.004 ± 0.070	*0.767*
Speed 10 m (s)	1.737 ± 0.077	1.718 ± 0.069	1.744 ± 0.061	1.724 ± 0.063	*0.981*
*t*-Test (s)	8.95 ± 0.32	9.05 ± 0.13	8.99 ± 0.35	9.02 ± 0.10	*0.348*
1RM Bench Press (kg)	87.22 ± 7.12	88.10 ± 8.53	87.27 ± 7.64	88.34 ± 9.07	*0.679*
1RM Back Squat (kg)	142.78 ± 19.86	131.44 ± 11.58	143.33 ± 19.60	131.41 ± 8.80	*0.800*

Data are presented as means ± SD. RMR: resting metabolic rate, **PRO-High PT**: Protein trial—High playing time group, **PRO-Mod PT**: Protein trial—Moderate Playing time group, **PLA-High PT**: Placebo trial—High playing time group, **PLA-Mod PT**: Placebo trial—Moderate playing time group. ^a^ Indicates significant difference vs. PRO-High PT (*p* < *0.05*). ^c^ Indicates significant difference vs. PLA-High PT (*p* < *0.05*).

### 2.3. Diet Monitoring and Supplementation

Before the adaptive period, in each trial, participants were asked to complete 5-day food diaries to assess their daily energy and macronutrient intake. Based on analysis of food diaries, they were provided with individualized dietary plans during the adaptive period that provided them with the recommended macronutrient intake [30], and were instructed to maintain them during the experimental trials. To ensure that they followed this instruction, participants provided a 24 h diet recall daily throughout the 6-day experimental period in each trial. Food diaries and recalls were analyzed with Science Fit Diet 200A (Science Technologies, Athens, Greece) by a registered dietitian.

Participants consumed either a protein supplement, which provided 80 g of milk protein (Milk Protein Smooth, MyProtein, Manchester, UK), or an isoenergetic placebo supplement (maltodextrin, MyProtein, Manchester, UK) in a random order. The supplementation protocol included 80 g of protein or maltodextrin on game days and 20 g of protein or maltodextrin on the two days after Game 3. On game days, supplements were consumed as repeated “pulsed” doses, with the first one administrated immediately post-game (30 g of protein/maltodextrin). The remaining two doses were provided once every 3 h on two occasions (25 g of protein/placebo at +3 h and 25 g of protein/placebo at +6 h) after the game. This supplementation scheme was previously shown to optimize muscle protein synthesis over a 12 h recovery period following resistance exercise [31,32]. On practice days, supplements were consumed as a single dose (20 g of protein/placebo) after performance testing. All drinks were isovolumetric (300–350 mL) and flavored with banana to make the contents indistinguishable and non-transparent [20]. Participants were asked daily if they knew what the drink was, and out of 160 responses, 146 responded, “I do not know,” 10 were incorrect guesses, and only 4 were correct guesses, probably by luck. Therefore, it is believed that the participants were well blinded.

### 2.4. Baseline Testing

#### 2.4.1. Anthropometrics and Body Composition

Body height was assessed using a wall-mounted stadiometer to the nearest 0.1 cm (SECA 206, SECA, Hamburg, Germany). Body weight was measured with a beam balance (flat scale SECA 806, SECA, Hamburg, Germany) to the nearest 0.1 kg. Body mass index (BMI) was calculated as body mass per height squared (kg/m^2^). Dual-energy X-ray absorptiometry (GE Healthcare, Lunar DPX-NT, Madison, WI, USA) was performed for body composition analysis, as previously described [26].

#### 2.4.2. Resting Metabolic Rate

RMR was assessed through indirect calorimetry (Fitmate Pro, Cosmed, Rome, Italy). Before measurement, participants were rested in a supine position for 30 min in a private laboratory room at a normal temperature, under dim lights, and in a state of quiescence. The canopy hood of FITMATE Pro was worn by participants; they then stayed awake for 20 min to complete the assessment. The first 5 min of each test were discarded, and the measurement continued until the steady state period when the RMR’s coefficient of variation (CV) was ≤10%. Calibration was performed before each measurement according to the manufacturer’s instructions [33].

#### 2.4.3. Agility and Speed Performance Assessment

Speed performance was assessed with 5 and 10 m sprints on a basketball court, according to the procedure of Chatzinikolaou et al. [6]. Agility was measured using the *t*-Test, also described by Chatzinikolaou et al. [6]. The time to complete speed and agility assessments was obtained by infrared light sensors (Newtest Oy, Oulu, Finland). Both speed and agility assessments were conducted during the baseline assessment periods.

#### 2.4.4. Lower-Limb Power

Lower-limb power was assessed using Countermovement Jump (CMJ) on a jump contact platform (Newtest Oy, Oulu, Finland), with jump heights calculated based on flight time, following the procedure described by Chatzinikolaou et al. [5].

#### 2.4.5. Strength Assessment

Lower- and upper-body submaximal strength was assessed bilaterally using the squat and bench press, both performed with a barbell and free weights. The 1-repetition Maximum (1 RM) was estimated using the 6–8 RM submaximal method [34]. Participants first completed a warm-up set of 15 repetitions at 50% of their typical 10-repetition training weight, followed by a set of 10 repetitions using their regular training load. Two additional sets were then used to identify the maximum weight they could lift for 6–8 repetitions. The 1 RM was calculated using the Epley equation [35].

#### 2.4.6. Basketball-Specific Conditioning Assessment

The basketball-specific conditioning was assessed with two intermittent tests. To assess intermittent endurance capacity, the YoYo intermittent endurance test was conducted during the baseline assessment period, as previously described by Papanikolaou et al. [36]. In addition, the YoYo Intermittent Recovery Level 2 (YoYo IR2) test was utilized to assess athletes’ ability to perform high-intensity repetitive exercise. This test evaluates the ability to perform intense intermittent exercise repeatedly with a high aerobic energy contribution while requiring significant anaerobic energy turnover. The YoYo IR2 test was conducted according to the protocol described by Krustrup et al. [37].

### 2.5. Study Outcomes

#### 2.5.1. Muscle Function

For isokinetic strength and maximal voluntary isometric contraction (MVIC) assessment, participants performed concentric and eccentric isokinetic peak torque tests at 60°/s and 180°/s, as well as maximal isometric strength assessments of the knee extensors (KE) and knee flexors (KF) on both the dominant and non-dominant limbs. After a 10 min warm-up on a cycle ergometer (Monark 834E, Monark Exercise AB, Vansbro, Sweden), participants received detailed instructions and completed a familiarization protocol with submaximal resistance on an isokinetic dynamometer (Cybex 6000, CSMi, Stoughton, MA, USA). The athlete’s positioning and the testing protocol followed the guidelines described by Draganidis et al. [38]. The same assessments were repeated daily during both trials, but only on the dominant leg of each participant. After data analysis, the functional ratio (FR) was calculated as the ratio of eccentric KE to concentric KF isokinetic peak force at both 60°/s and 180°/s [39].

#### 2.5.2. Blood Sampling and Assays

After an overnight fast, blood samples (~14 mL) were collected via venipuncture from the antecubital vein while participants were seated. Blood intended for CK measurement was collected in tubes containing ethylenediaminetetraacetic acid (EDTA) and centrifuged at 1370× *g* at four °C for 10 min to separate the plasma. CK was determined spectrophotometrically using a commercially available kit (Spinreact, Sant Esteve, Spain). For the analysis of reduced glutathione (GSH), packed erythrocytes were obtained after plasma separation and subsequently lysed [40]. The lysate was used for the GSH assay, which was performed spectrophotometrically following the addition of 5,5-dithiobis-2-nitrobenzoic acid (DTNB) to the sample. All samples were stored at −75 °C until analysis and assays were conducted in duplicate to ensure accuracy.

### 2.6. Statistical Analyses

Data are presented as means ± SE. A one-sample Kolmogorov–Smirnoff test was used to determine data normality (since data normality was verified, a nonparametric test was not necessary). To ensure study accuracy, body composition and performance were examined before each trial, and diet was monitored during the trials. Two-way repeated measures analysis of variance (ANOVA) was used to investigate possible differences. Time (pre-first and pre-second trials) and group (moderate and high playing time) were the two factors. Based on the experimental design, three factors are recognized: time represents the time point of dependent variable evaluations (Pre G1, Pre G2, 24 h Post G3, 48 h Post G3, 72 h Post G3); group, which is based on participation time with two levels (moderate and high); and trial, which represents the consumption of protein or placebo. Therefore, a three-way (time × group × trial) repeated measures ANOVA with planned contrasts on different time points was used to analyze the data for isokinetic performance and indirect muscle damage markers. The interaction of three factors was examined at first. If no interaction was detected, then two-factor interaction was examined. If no two-factor interactions were detected, then the main effects of trial, participating time, and time were examined. A Bonferroni correction analysis was utilized for pairwise comparisons when a significant interaction or main effect was observed. Significance was accepted at *p* < 0. 05. SPSS was used for all analyses (SPSS for Windows, version 29.0, Chicago, IL, USA).

## 3. Results

**Figure 2:**
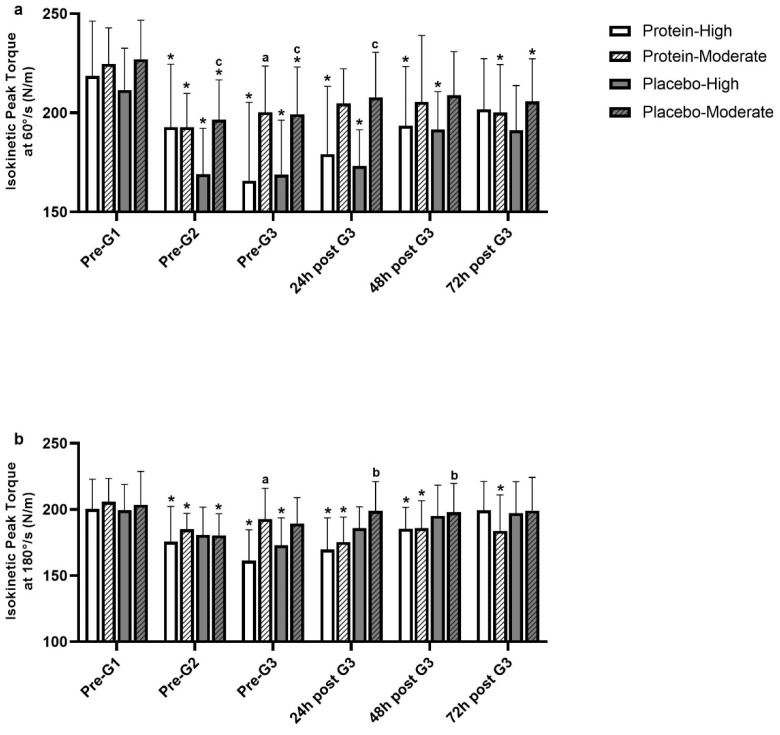
Changes in eccentric peak torque of the knee flexors at 60°/s (**a**) and 180°/s (**b**) in High PT and Mod PT groups, in PLA and PRO. Data are presented as mean ± SD. PRO-High PT: Protein trial—High playing time group, PRO-Mod PT: Protein trial—Moderate Playing time group, PLA-High PT: Placebo trial—High playing time group, PLA-Mod PT: Placebo trial—Moderate playing time group. * Indicates significant difference vs. Pre-G1 (*p* < *0.05*). ^a^ Indicates significant difference vs. PRO-High PT (*p* < *0.05*). ^b^ Indicates significant difference vs. PRO-Mod PT (*p* < *0.05*). ^c^ Indicates significant difference vs. PLA-High PT (*p* < *0.05*).

**Figure 3:**
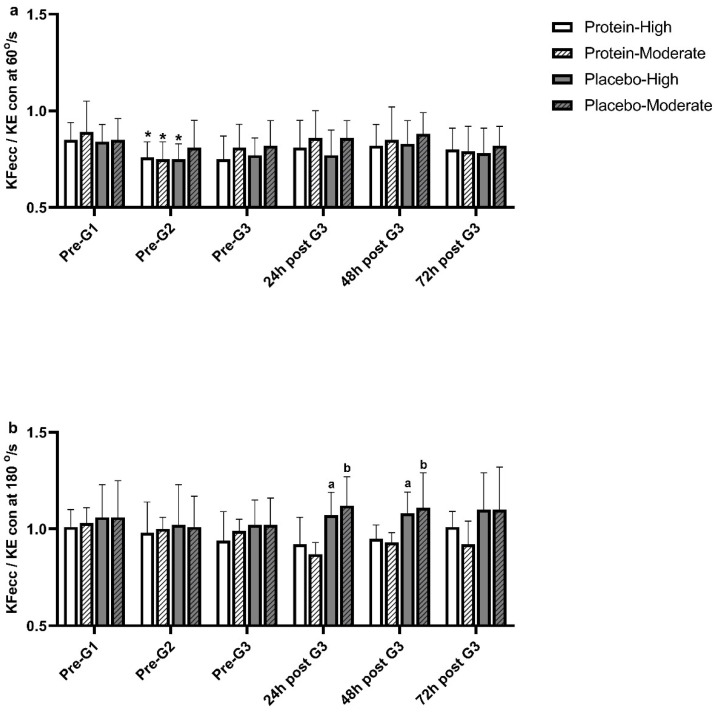
Changes in functional ratio at 60°/s (**a**) and 180°/s (**b**) in High PT and Mod PT groups, in PLA and PRO. Data are presented as mean ± SD. **PRO-High PT**: Protein trial—High playing time group, **PRO-Mod PT**: Protein trial—Moderate Playing time group, **PLA-High PT**: Placebo trial—High playing time group, **PLA-Mod PT**: Placebo trial—Moderate playing time group. * Indicates significant difference vs. Pre-G1 (*p* < *0.05*). ^a^ Indicates significant difference vs. PRO-High PT (*p* < *0.05*). ^b^ Indicates significant difference vs. PRO-Mod PT (*p* < *0.05*).

**Figure 4:**
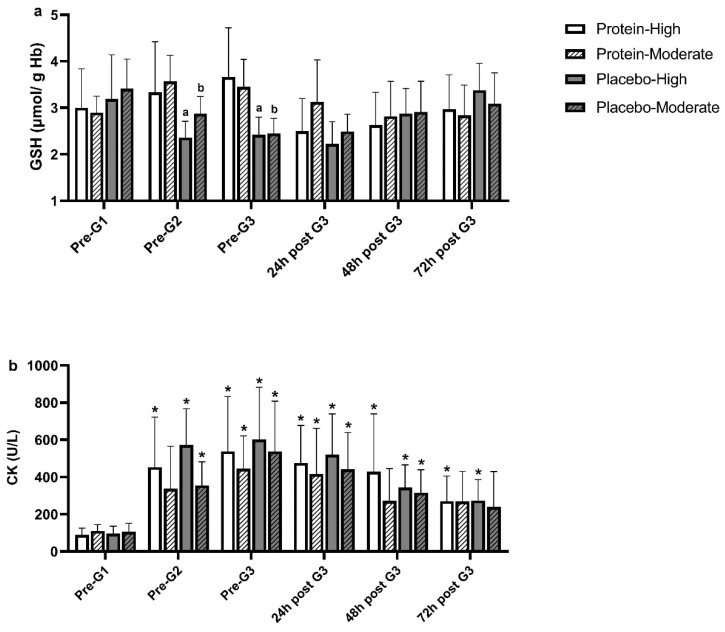
Changes in erythrocyte GSH concentration (**a**) and serum CK activity (**b**) in High PT and Mod PT groups, in PLA and PRO. Data are presented as mean ± SD. **PRO-High PT**: Protein trial—High playing time group, **PRO-Mod PT**: Protein trial—Moderate Playing time group, **PLA-High PT**: Placebo trial—High playing time group, **PLA-Mod PT**: Placebo trial—Moderate playing time group. * Indicates significant difference vs. Pre-G1 (*p* < *0.05*). ^a^ Indicates significant difference vs. PRO-High PT (*p* < *0.05*). ^b^ Indicates significant difference vs. PRO-Mod PT (*p* < *0.05*).

This section details the study’s findings, organized by main areas: participants’ characteristics, dietary intake, muscle function, torque measurements, muscle strength ratios, muscle damage, and redox status indices. Tables and figures summarize the results between High PT and Mod PT groups across PRO and PLA trials. Table 1 presents detailed participants’ characteristics, while dietary intake during the experimental period, shown in Table 2, highlights changes in energy, protein, and amino acid intake across game and recovery days. Muscle function is assessed through isometric strength of the knee KF and KE, torque measurements including concentric peak torque of KF and KE, and eccentric peak torque of KE at 60°/s and 180°/s. Muscle strength ratios, including functional and conventional ratios at 60°/s and 180°/s, are analyzed across trials and groups, with results displayed in Table 3, Table 4 and Table 5 and Figure 2 and Figure 3. Lastly, indices of redox status and muscle damage, including erythrocyte GSH concentration and serum CK activity, are presented in Figure 4.

### 3.1. Participants’ Characteristics

Table 1 provides a detailed presentation of participants’ characteristics in each group [High Playing Time (High PT) vs. Moderate Playing Time (Mod PT)] prior to each trial [Protein trial (PRO) vs. Placebo trial (Pla)]. There were no differences between groups and trials in participants’ characteristics or any of the outcome variables before Game 1 (Pre-G1). As expected, the average playing time (of the three games) differed significantly between groups (*p* < *0.05*), with participants in High PT playing almost 30 min while those in Mod PT participated for ~15 min in each game in both trials.

### 3.2. Dietary Intake

Participants’ dietary intake during the experimental period in PLA and PRO trials is shown in Table 2. Despite some normal day-to-day fluctuations, no significant changes were noted in total energy intake over time. All treatment groups had similar energy (kcal) intake, ranging from 2165 kcal (±219 kcal) to 2623 kcal (±480 kcal) per day. Total protein intake increased from 1.2–1.3 g/kg/day (protein intake in PLA) to 2.1–2.3 g/kg/day on game days (Days 1–3) and to 1.5–1.6 g/kg/day on recovery days post-G3 (Days 4 and 5) in the PRO trial. Of note, supplementation with protein also resulted in a remarkable increase in the intake of total cysteine (from 1.4–1.7 g/day to 8.7–8.9 g/day on game days and to 3.2–3.3 g/day on recovery days), glutamic acid (from 21.6–24.2 g/day to 37.5–40.0 g/day on game days and to 23.8–25.9 g/day on recovery days), and glycine (from 2.0–2.1 g/day to 3.4–3.5 g/day on game days and to 2.4 g/day on recovery days) throughout the experimental period. In the PLA trial, total carbohydrate intake increased from 221–279 g/day (intake in the PRO trial) to 302–358 g/day on game days and 260–301 g/day on recovery days. Total fat intake remained unaltered over time and similar between treatment groups.

**Table 2:** Participants’ dietary intake during the experimental period in PLA and PRO trials.

	Day 1 G1-Day	Day 2 G2-Day	Day 3 G3-Day	Day 4 24 h Post-G3	Day 5 48 h Post-G3
**Total energy (kcal/day)**					
PRO—High PT	2414.37 ± 276.74	2305.52 ± 340.83	2606.76 ± 444.15	2427.99 ± 357.24	2207.10 ± 248.3
PRO—Mod PT	2492.48 ± 402.45	2588.26 ± 504.53	2596.18 ± 462.28	2358.91 ± 403.02	2291.71 ± 240.72
PLA—High PT	2371.58 ± 272.08	2359.03 ± 301.11	2541.9 ± 490.59	2389.41 ± 359.51	2164.52 ± 218.76
PLA—Mod PT	2485.36 ± 414.45	2618.98 ± 520.03	2622.66 ± 479.63	2369.12 ± 425.97	2313.09 ± 224.80
**Protein (g/day)**					
PRO—High PT	191.63 ± 18.82	178.74 ± 22.48	188.59 ± 7.8	131.17 ± 11.17 *	129.26 ± 10.77 *
PRO—Mod PT	189.88 ± 27.03	191.84 ± 32.53	178.98 ± 31.4	132.12 ± 21.56 *	128.18 ± 21.64 *
PLA—High PT	111.56 ± 16.71 ^a^	103.76 ± 17.56 ^a^	104.05 ± 6.91 ^a^	103.25 ± 10.56 ^a^	105.84 ± 8.39 ^a^
PLA—Mod PT	109.78 ± 26.53 ^b^	110.64 ± 29.08 ^b^	103.82 ± 28.12 ^b^	109.1 ± 22.69 ^b^	105.87 ± 21.07 ^b^
**Protein (g/kg BW/day)**					
PRO—High PT	2.26 ± 0.34	2.11 ± 0.32	2.23 ± 0.26	1.55 ± 0.2 *	1.52 ± 0.15 *
PRO—Mod PT	2.14 ± 0.39	2.16 ± 0.43	2.02 ± 0.41	1.49 ± 0.26 *	1.44 ± 0.24 *
PLA—High PT	1.32 ± 0.26 ^a^	1.22 ± 0.21 ^a^	1.23 ± 0.18 ^a^	1.22 ± 0.18 ^a^	1.23 ± 0.15 ^a^
PLA—Mod PT	1.23 ± 0.31 ^b^	1.24 ± 0.34 ^b^	1.17 ± 0.33 ^b^	1.23 ± 0.28 ^b^	1.19 ± 0.23 ^b^
**Carbohydrate (g/day)**					
PRO—High PT	240.02 ± 36.67	220.57 ± 35.84	232.95 ± 31.29	252.28 ± 47.92	240.33 ± 61.9
PRO—Mod PT	266.36 ± 65.63	262.84 ± 56.55	275.71 ± 57.91	278.7 ± 55.76	258.06 ± 63.86
PLA—High PT	315.36 ± 24.68 ^a^	301.96 ± 34.23 ^a^	303.58 ± 43.46 ^a^	280.44 ± 47.86 *^,a^	260.14 ± 57.02 *^,a^
PLA—Mod PT	343.95 ± 64.29 ^b^	347.67 ± 59.64 ^b^	357.51 ± 57.64 ^b^	301.14 ± 55.57 *^,b^	283.44 ± 64.74 *^,b^
**Fat (g/day)**					
PRO—High PT	76.42 ± 26.05	78.70 ± 20.57	102.29 ± 45.28	99.35 ± 39.31	79.86 ± 15.45
PRO—Mod PT	74.17 ± 16.84	85.5 ± 27.79	86.38 ± 27.1	79.51 ± 21.91	82.97 ± 16.49
PLA—High PT	73.76 ± 25.77	81.80 ± 22.71	101.26 ± 46.91	94.96 ± 36.92	78.62 ± 17.38
PLA—Mod PT	74.49 ± 18.7	87.3 ± 28.59	86.37 ± 27.26	80.9 ± 23.27	83.98 ± 16.86
**Cysteine (g/day)**					
PRO—High PT	8.73 ± 0.54	8.68 ± 0.56	8.86 ± 0.59	3.22 ± 0.4 *	3.25 ± 0.57 *
PRO—Mod PT	8.78 ± 0.59	8.81 ± 0.63	8.63 ± 0.67	3.32 ± 0.68 *	3.18 ± 0.57 *
PLA—High PT	1.69 ± 0.56 ^a^	1.46 ± 0.59 ^a^	1.72 ± 0.61 ^a^	1.44 ± 0.41 ^a^	1.41 ± 0.57 ^a^
PLA—Mod PT	1.64 ± 0.65 ^b^	1.61 ± 0.62 ^b^	1.45 ± 0.7 ^b^	1.55 ± 0.72 ^b^	1.38 ± 0.57 ^b^
**Glutamic Acid (g/day)**					
PRO—High PT	39.96 ± 7.18	37.74 ± 8.11	39.28 ± 6.93	24.43 ± 6.05 *	24.73 ± 7.74 *
PRO—Mod PT	38.96 ± 9.22	39.44 ± 6.62	37.47 ± 10.1	25.85 ± 11.67 *	23.83 ± 7.26 *
PLA—High PT	25.1 ± 7.69 ^a^	22.56 ± 8.71 ^a^	24.17 ± 7.54 ^a^	20.59 ± 6.43 ^a^	20.86 ± 7.91 ^a^
PLA—Mod PT	23.62 ± 9.4 ^b^	23.15 ± 9.37 ^b^	21.58 ± 10.74 ^b^	22.3 ± 12.18 ^b^	20.27 ± 7.53 ^b^
**Glycine (g/day)**					
PRO—High PT	3.39 ± 0.34	3.44 ± 0.28	3.49 ± 0.24	2.39 ± 0.28 *	2.41 ± 0.24 *
PRO—Mod PT	3.41 ± 0.25	3.44 ± 0.25	3.51 ± 0.27	2.42 ± 0.17 *	2.41 ± 0.17 *
PLA—High PT	2.03 ± 0.33 ^a^	2.06 ± 0.29 ^a^	2.1 ± 0.24 ^a^	2.06 ± 0.27 ^a^	2.05 ± 0.24 ^a^
PLA—Mod PT	2.03 ± 0.26 ^b^	2.05 ± 0.25 ^b^	2.13 ± 0.28 ^b^	2.09 ± 0.19 ^b^	2.06 ± 0.18 ^b^

Data are presented as means ± SD. **PRO-High PT**: Protein trial—High playing time group, **PRO-Mod PT**: Protein trial—Moderate Playing time group, **PLA-High PT**: Placebo trial—High playing time group, **PLA-Mod PT**: Placebo trial—Moderate playing time group. * Indicates significant difference vs. Day 1 in the same group (*p < 0.05*). ^a^ Indicates significant difference vs. PRO-High PT (*p* < *0.05*). ^b^ Indicates significant difference vs. PRO-Mod PT (*p* < *0.05*).

### 3.3. Performance

#### 3.3.1. Isometric Strength

No trial or group effect was observed in the isometric strength of either KE or KF in the dominant limb (Table 3). In KE, isometric strength declined significantly at Pre-G2 (PRO-High PT: −8%, *p* = *0.007–0.043*; PRO-Mod PT: −8–10%, *p* = *0.003–0.014*) as compared to Pre-G1, demonstrating its lowest values at 24 h post-G3 (PRO-High PT: −9–13%, *p* < *0.001*; PRO-Mod PT: −8–9%, *p* = *0.002–0.006*) in both PRO-High PT and PRO-Mod PT groups. However, it recovered at 48 h post-G3 and 72 h post-G3 in Mod PT and High PT groups, respectively and independently of the trial, indicating that increasing the playing time might delay the recovery of isometric strength in KE. In KF, isometric strength was significantly reduced at Pre-G2 (PRO-High PT: −10–13%, *p* = *0.000–0.031*; PRO-Mod PT: −5–11%, *p* = *0.014–0.033*), displayed its lowest values at Pre-G3 (PRO-High PT: −17–14%, *p* < *0.001*; PRO-Mod PT: −8–14%, *p* = *0.028–0.033*), and remained below Pre-G1 values throughout the recovery period in both PRO-High PT and PRO-Mod PT groups.

**Table 3:** Changes in isometric strength of the knee extensors and flexors in the dominant limb.

	Pre-G1	Pre-G2	Pre-G3	24 h Post-G3	48 h Post-G3	72 h Post-G3
**Isometric Strength (Nm)—Knee Extensors**						
PRO—High PT	310.22 ± 51.58	285.77 ± 48.80 *	270.57 ± 42.87 *	270.88 ± 47.83 *	284.89 ± 42.39 *	299.22 ± 59.34
PRO—Mod PT	316.63 ± 26.26	290.16 ± 20.40 *	290.80 ± 27.79 *	287.44 ± 34.96 *	291.86 ± 30.69	299.14 ± 40.95
PLA—High PT	306.97 ± 52.85	281.22 ± 46.35 *	282.56 ± 52.42 *	278.59 ± 51.79 *	279.47 ± 52.42 *	296.60 ± 49.33
PLA—Mod PT	306.83 ± 35.79	277.32 ± 28.85 *	284.62 ± 37.02 *	283.41 ± 30.45 *	292.62 ± 28.98	294.77 ± 31.22
**Isometric Strength (Nm)—Knee Flexors**						
PRO—High PT	193.46 ± 26.58	174.24 ± 22.51 *	160.73 ± 18.64 *	166.51 ± 19.92 *	175.54 ± 30.38 *	175.56 ± 24.17 *
PRO—Mod PT	194.47 ± 15.23	173.27 ± 12.75 *	168.32 ± 21.43 *	173.72 ± 21.79 *	179.42 ± 20.15 *	177.83 ± 25.21 *
PLA—High PT	185.00 ± 21.34	161.06 ± 23.87 *	160.10 ± 22.91 *	163.74 ± 14.51 *	172.48 ± 22.19 *	171.17 ± 26.62 *
PLA—Mod PT	191.29 ± 9.88	181.54 ± 11.75	175.59 ± 15.03 *	175.89 ± 20.03 *	182.77 ± 15.85	177.33 ± 11.80 *

Data are presented as means ± SD. **PRO-High PT**: Protein trial—High playing time group, **PRO-Mod PT**: Protein trial—Moderate Playing time group, **PLA-High PT**: Placebo trial—High playing time group, **PLA-Mod PT**: Placebo trial—Moderate playing time group. * Indicates significant difference vs. Pre-G1 (*p* < *0.05*).

#### 3.3.2. Concentric Peak Torque

Changes in concentric peak torque of the knee extensors and flexors in PRO and PLA trials are shown in Table 4. In the High PT group, concentric peak torque of the knee extensors at 60°/s declined at pre-G3 (−14%, *p* = *0.006*) and 24 h post-G3 (−13%, *p* = *0.002*) and recovered gradually thereafter, in the PRO trial, whereas in PLA, it reduced at pre-G2 (−11%, *p* = *0.001*) and remained below pre-G1 values until 48 h post-G3 (by 13–8%, *p* = *0.000–0.019*). A significant difference between trials was observed at pre-G2, where participants’ concentric peak torque was lower in the PLA trial by ~11% (PLA: 226.91 ± 21.70 Nm vs. PRO: 254.11 ± 35.99 Nm, *p* < *0.001*). Likewise, in the Mod PT group, protein supplementation induced a protective effect on knee extensors’ concentric peak torque, as no changes over time were observed in the PRO trial, while in PLA, a significant reduction was noted at 24 h (−9%, *p* = *0.009*) and 48 h (−11%, *p* = *0.007*) post-G3.

Although no significant differences between trials were observed in the High PT group, concentric peak torque of the knee extensors at 180°/s decreased at pre-G2 (~4%, *p* = *0.035*), pre-G3 (~4%, *p* = *0.011*), and 24 h post-G3 (~4%, *p* = *0.001*) compared to pre-G1 in PRO, whereas in PLA it remained unaltered over time. In contrast, in the Mod PT group, concentric peak torque (of the knee extensors at 180°/s) declined by 9–8% (*p* = *0.002–0.016*) from pre-G2 to 48 h post-G3 in PLA, while in PRO, a significant reduction compared to pre-G1 was noted only at 24 h post-G3 (−5%, *p* = *0.030*). Furthermore, no significant differences were observed between the trials (*p* = 0.576).

**Table 4:** Changes in concentric peak torque of the knee extensors and flexors in the dominant limb.

	Pre-G1	Pre-G2	Pre-G3	24 h Post-G3	48 h Post-G3	72 h Post-G3
**Concentric Peak Torque (Nm) at 60** °**/s—*Knee Extensors***						
**PRO—High PT**	257.72 ± 32.11	254.11 ± 35.99	221.22 ± 43.43 *	223.28 ± 41.50 *	238.46 ± 30.72	253.50 ± 29.48
**PRO—Mod PT**	258.73 ± 39.38	260.57 ± 40.62	252.53 ± 36.53	242.54 ± 36.47	245.24 ± 38.45	256.99 ± 40.07
**PLA—High PT**	254.16 ± 34.42	226.91 ± 21.70 *^,a^	222.10 ± 36.44 *	227.73 ± 32.48 *	232.87 ± 29.02 *	248.66 ± 37.52
**PLA—Mod PT**	269.53 ± 36.91	247.56 ± 37.31	247.83 ± 38.53	244.44 ± 34.78 *	239.68 ± 30.64 *	254.68 ± 31.92
**Concentric Peak Torque (Nm) at 180** °**/s—*Knee Extensors***						
**PRO—High PT**	190.27 ± 24.51	182.16 ± 28.01 *	171.56 ± 23.50 *	175.88 ± 26.90 *	180.68 ± 18.81	181.39 ± 22.01
**PRO—Mod PT**	193.66 ± 19.99	186.22 ± 23.18	192.42 ± 20.50	183.69 ± 20.34 *	185.06 ± 21.55	187.67 ± 22.64
**PLA—High PT**	186.22 ± 22.32	174.77 ± 17.45	176.08 ± 19.83	177.60 ± 19.72	175.90 ± 19.47	181.10 ± 19.06
**PLA—Mod PT**	200.14 ± 20.58	182.83 ± 27.01 *	184.83 ± 20.56 *	181.25 ± 22.26 *	181.50 ± 20.58 *	191.66 ± 18.35
**Concentric Peak Torque (Nm) at 60** °**/s—*Knee Flexors***						
**PRO—High PT**	171.17 ± 19.17	161.86 ± 20.62	149.58 ± 19.74 *	151.53 ± 25.43 *	163.01 ± 23.22	168.63 ± 24.91
**PRO—Mod PT**	173.80 ± 14.87	157.83 ± 12.47 *	166.17 ± 15.34	158.82 ± 15.89	155.19 ± 12.17	163.62 ± 14.71
**PLA—High PT**	171.87 ± 16.62	152.17 ± 18.74 *	150.26 ± 18.56 *	152.69 ± 16.17 *	157.86 ± 23.40	156.81 ± 16.94
**PLA—Mod PT**	174.16 ± 17.04	169.02 ± 18.49	163.53 ± 16.72	158.46 ± 17.25 *	154.30 ± 17.08	161.86 ± 14.13
**Concentric Peak Torque (Nm) at 180** °**/s—*Knee Flexors***						
**PRO—High PT**	138.31 ± 17.98	127.40 ± 23.01	116.70 ± 17.39	120.34 ± 17.99	120.47 ± 12.33	137.76 ± 16.22
**PRO—Mod PT**	147.42 ± 13.79	131.84 ± 14.21	130.49 ± 16.82	131.30 ± 15.64	148.33 ± 18.67 ^a^	145.83 ± 20.64
**PLA—High PT**	137.67 ± 19.26	120.44 ± 10.76	119.17 ± 13.39	114.59 ± 8.32	128.26 ± 22.19	133.89 ± 18.75
**PLA—Mod PT**	145.32 ± 17.92	132.59 ± 23.53	133.68 ± 16.26	135.48 ± 18.11 ^c^	130.87 ± 16.02 ^b^	137.58 ± 19.89

Data are presented as means ± SD. **PRO-High PT**: Protein trial—High playing time group, **PRO-Mod PT**: Protein trial—Moderate Playing time group, **PLA-High PT**: Placebo trial—High playing time group, **PLA-Mod PT**: Placebo trial—Moderate playing time group. * Indicates significant difference vs. Pre-G1 (*p* < *0.05*). ^a^ Indicates significant difference vs. PRO-High PT (*p* < *0.05*). ^b^ Indicates significant difference vs. PRO-Mod PT (*p* < *0.05*). ^c^ Indicates significant difference vs. PLA-High PT (*p* < *0.05*).

Concentric peak torque of the knee flexors at 60°/s in the High PT group decreased at pre-G3 (−13%, *p* < *0.001*) and 24 h post-G3 (−11%, *p* = *0.025*) in PRO, and similar reductions were evident in PLA at pre-G2 (−11%, *p* = *0.003*), pre-G3 (−13%, *p* = *0.009*) and 24 h post-G3 (−11%, *p* = *0.005*). In the Mod PT group, a reduction was observed only at pre-G2 in PRO (−9%, *p* = *0.001*) and at 24 h post-G3 in PLA (−9%, *p* < *0.001*).

Concentric peak torque of the knee flexors at 180°/s remained unaffected over time in both groups and trials. However, when groups were compared, concentric peak torque (of the knee flexors at 180°/s) was higher in Mod PT than High PT at 48 h post-G3 in PRO (Mod PT: 148.33 ± 18.67 Nm vs. High PT: 120.47 ± 12.33 Nm, *p* = *0.002*) and at 24 h post-G3 in PLA (Mod PT: 135.48 ± 18.11 Nm vs. High PT: 114.59 ± 8.32 Nm, *p* = *0.006*). The comparison between trials revealed higher concentric peak torque (of the knee flexors at 180°/s) in PRO compared to PLA at 48 h post-G3 in the Mod PT group (PRO: 148.33 ± 18.67 Nm vs. PLA: 130.87 ± 16.02 Nm, *p* = *0.019*).

#### 3.3.3. Eccentric Peak Torque of Knee Flexors

Changes in eccentric peak torque of the knee flexors at 60°/s and 180°/s in PRO and PLA trials are shown in Figure 2. Eccentric peak torque of knee flexors at 60°/s in the High PT group decreased at pre-G2 from 218.5 ± 27.8 Nm to 192.8 ± 31.7 Nm (−12%, *p* = *0.004*) and from 211.4 ± 21.2 Nm to 169.6 ± 23.1 Nm (−20%, *p* = *0.004*) in PRO and PLA trials, respectively. It displayed its lowest values at pre-G3 in both trials (PRO: 165.8 ± 39.5 Nm, *p* < *0.001*; PLA: 168.9 ± 27.4 Nm, *p* < *0.001*) and remained below pre-G1 values at 24 h (PRO: 179.12 ± 32.3 Nm, *p* < *0.001*; PLA: 173.2 ± 18.3 Nm, *p* = *0.003*) and 48 h post-G3 (PRO: 193.5 ± 29.9 Nm, *p* = *0.004*; PLA: 191.6 ± 19.1 Nm, *p* = *0.048*). In the Mod PT group, eccentric peak torque (of knee flexors at 60°/s) declined at pre-G2 (−13%, *p* = *0.004* and pre-G3 (−12%, *p* = *0.008*) in PLA and at pre-G2 (−14%, *p* < *0.001*) in PRO, and recovered after that. Between-groups comparison revealed lower eccentric peak torque (at 60°/s) in High PT compared to Mod PT at pre-G2, pre-G3, and 24 h post-G3 by 16% (*p* = *0.16*), 18% (*p* = *0.023*), and 20% (*p* = *0.003*), respectively, in the PLA trial. In PRO, eccentric peak torque was lower in High PT compared to Mod PT only at pre-G3, by 17% (*p* = *0.038*).

In the High PT group, eccentric peak torque of knee flexors at 180°/s declined by ~12% (*p* = *0.001*) at pre-G2, reached its lowest value at pre-G3 (−19%, *p* = *0.001*), and remained reduced up to 48 h post-G3 (24 h post-G3: −15%, *p* = *0.007*; 48 h post-G3: −7%, *p* = *0.007*) in PRO. Unexpectedly, in PLA, eccentric peak torque (at 180°/s) decreased only at pre-G3 (−13%, *p* = *0.005*), but no significant differences were observed between trials in the High PT group. Likewise, in the Mod PT group, eccentric peak torque (at 180°/s) decreased throughout the recovery period in PRO (*p* = *0.033*), whereas in PLA, it was impaired only at pre-G2 (*p* = *0.033*). Of note, PLA demonstrated increased eccentric peak torque (at 180°/s) compared to PRO at 24 h (PLA: 199.08 ± 22.02 vs. PRO: 175.39 ± 18.92, *p* = *0.007*) and 48 h (PLA: 198.07 ± 21.64 vs. PRO: 185.81 ± 20.88, *p* = *0.027*) post-G3 in the Mod PT group. A group effect was evident in the PRO trial at pre-G3, where eccentric peak torque (at 180°/s) was lower by 20% (*p* = *0.011*) in the High PT compared to the Mod PT group.

#### 3.3.4. Conventional Ratio

Table 5 presents changes in conventional ratio at 60°/s and 180°/s in PRO and PLA trials. The conventional ratio at 60°/s was not affected over time either by playing time or by supplementation. At 180°/s, the conventional ratio remained unaffected over time in PRO but declined at pre-G3 (−8%, *p* = *0.010*) and 24 h post-G3 (−12%, *p* = *0.036*) in PLA, in the High PT group. In the Mod PT group, the conventional ratio (at 180°/s) remained unchanged during recovery in PLA, whereas in PRO, it was reduced by 11% (*p* = *0.020*) at pre-G3. However, PRO demonstrated a higher conventional ratio (at 180°/s) than PLA at 48 h (PRO: 0.81 ± 0.12 vs. PLA: 0.72 ± 0.08, *p* = *0.027*) and 72 h (PRO: 0.78 ± 0.10 vs. PLA: 0.72 ± 0.09, *p* = *0.041*) post-G3 in the Mod PT group.

**Table 5:** Changes in conventional ratio at 60°/s and 180°/s.

	Pre-G1	Pre-G2	Pre-G3	24 h Post-G3	48 h Post-G3	72 h Post-G3
**Conventional ratio at 60** °**/s**						
**Pro—High PT**	0.6677 ± 0.0649	0.6420 ± 0.0845	0.6995 ± 0.1676	0.6883 ± 0.1148	0.6877 ± 0.0948	0.6659 ± 0.0785
**Pro—Mod PT**	0.6875 ± 0.1400	0.6199 ± 0.1226	0.6671 ± 0.0912	0.6599 ± 0.0466	0.6424 ± 0.0845	0.6445 ± 0.0641
**Pla—High PT**	0.6876 ± 0.1202	0.6717 ± 0.0725	0.6844 ± 0.0884	0.6773 ± 0.0847	0.6809 ± 0.0968	0.6389 ± 0.0829
**Pla—Mod PT**	0.6538 ± 0.0829	0.6926 ± 0.0980	0.6664 ± 0.0645	0.6543 ± 0.0758	0.6472 ± 0.0597	0.6430 ± 0.0856
**Conventional ratio at 180** °**/s**						
**Pro—High PT**	0.7297 ± 0.0700	0.7013 ± 0.0835	0.6809 ± 0.0563	0.6860 ± 0.0558	0.6702 ± 0.0707	0.7649 ± 0.0957
**Pro—Mo PT**	0.7655 ± 0.0812	0.7141 ± 0.0900	0.6853 ± 0.1168 *	0.7166 ± 0.0671	0.8085 ± 0.1197	0.7809 ± 0.1021
**Pla—High PT**	0.7393 ± 0.0608	0.6909 ± 0.0424	0.6783 ± 0.0477 *	0.6491 ± 0.0493 *	0.7266 ± 0.0807	0.7393 ± 0.0717
**Pla—Mod PT**	0.7263 ± 0.0548	0.7271 ± 0.0903	0.7256 ± 0.0710	0.7525 ± 0.0940	0.7240 ± 0.0783 ^b^	0.7187 ± 0.0848 ^b^

Data are presented as means ± SD. **PRO-High PT**: Protein trial—High playing time group, **PRO-Mod PT**: Protein trial—Moderate Playing time group, **PLA-High PT**: Placebo trial—High playing time group, **PLA-Mod PT**: Placebo trial—Moderate playing time group. * Indicates significant difference vs. Pre-G1 (*p* < *0.05*). ^b^ Indicates significant difference vs. PRO-Mod PT (*p* < *0.05*).

#### 3.3.5. Functional Ratio

The functional ratio (KFecc/KEcon) at 180°/s remained unaffected over time in both groups and trials (Figure 3). However, KFecc/KEcon at 180°/s was greater in PLA compared to PRO at 24 h (High PT: PLA: 1.07 ± 0.12 vs. PRO: 0.92 ± 0.14, *p* = *0.042*; Mod PT: PLA: 1.12 ± 0.15 vs. PRO: 0.87 ± 0.06, *p* = *0.003*) and 48 h (High PT: PLA: 1.08 ± 0.11 vs. PRO: 0.95 ± 0.07, *p* = *0.038*; Mod PT: PLA: 1.10 ± 0.18 vs. PRO: 0.93 ± 0.05, *p* = *0.006*) post-G3 in both groups (Figure 3).

### 3.4. Blood Redox Status and Muscle Damage Indices

Changes in erythrocyte GSH concentration and serum CK activity are presented in Figure 4. Erythrocyte GSH concentration remained unaltered over time in both groups and trials. However, when trials were compared, erythrocyte GSH concentration was higher in PRO compared to PLA at pre-G2 (High PT: PRO: 3.33 ± 1.09 μmol/g Hb vs. PLA: 2.36 ± 0.35 μmol/g Hb, *p* = *0.020*; Mod PT: PRO: 3.57 ± 0.56 μmol/g Hb vs. PLA: 2.87 ± 0.37 μmol/g Hb, *p* = *0.035*) and pre-G3 (High PT: PRO: 3.66 ± 1.06 μmol/g Hb vs. PLA: 2.42 ± 0.38 μmol/g Hb, *p* = *0.047*; Mod PT: PRO: 3.45 ± 0.59 μmol/g Hb vs. PLA: 2.45 ± 0.32 μmol/g Hb, *p* = *0.019*) in both groups.

CK activity was markedly elevated at pre-G2 (PRO: from 89.2 ± 36.2 to 452.8 ± 269.0 U/L, *p* = *0.002*; PLA: from 96.5 ± 40.0 to 572.5 ± 194.7 U/L, *p* < *0.001*), peaked at pre-G3 (PRO: 537.8 ± 294.9 U/L, *p* < *0.001*; PLA: 602.8 ± 279.6 U/L, *p* < *0.001*) and remained above pre-G1 levels throughout recovery in the High PT group, with no differences between trials. In the Mod PT group, CK activity increased at pre-G2 (from 105.9 ± 45.6 to 355.3 ± 127.0 U/L. *p* = *0.002*), demonstrated its peak at pre-G3 (537.7 ± 270.0 U/L, *p* = *0.002*), and remained elevated up to 48 h post-G3 (316.4 ± 123.3 U/L, *p* = *0.001*) in PLA, while in PRO it increased at pre-G3 (from 110.7 ± 34.2 to 445.8 ± 175.4 U/L, *p* = *0.005*) and 24 h post-G3 (from 110.7 ± 34.2 to 416.3 ± 246.2 U/L, *p* = *0.006*).

## 4. Discussion

In this study, we assessed the impact of protein supplementation on muscle function recovery during a congested schedule consisting of three consecutive basketball games. In addition, to examine whether playing time affects muscle function responses to increased protein intake, players were allocated to a High PT or a Moderate PT group, participating for, on average, 30 min or 15 min, respectively, in each game. The primary findings were that (1) participation in three basketball games in a row results in muscle function deterioration lasting up to 48 h after the third game, (2) higher participation time induces a more pronounced effect on muscle trauma and function indices than a moderate participation time, and (3) increased protein intake on game days preserves erythrocyte GSH concentration but does not accelerate recovery of muscle function.

Previous research has revealed that participation in a single basketball game induces an inflammatory response and performance deterioration lasting for 24–48 h [6]. These responses are milder and last for a shorter time period compared to those induced by a soccer game, where players need more than 72 h to fully recover [41,42,43]. However, congested schedules in basketball are very common and might consist of either three games performed with a 48 h recovery period in between, especially in club competitions such as EuroLeague, NBA, or even domestic leagues [1,11], or three games performed in three consecutive days, a pattern that is usually applied in tournaments, lower-division leagues or national cup competitions, and U18 tournaments [11,12]. Therefore, information on how players can perform and recover during a congested schedule and whether specific nutritional strategies can elicit beneficial effects is of paramount significance for players, coaches, trainers, and sports nutritionists.

In the present study, participation in three consecutive games induced an almost 6-fold increase in serum CK concentration on game days, which remained elevated until 72 h post-G3 (by 3-fold) in the High PT group. In fact, at 24 h following each game, CK ranged between 500 and 600 IU, a response that is similar to that observed at 24 h following strenuous muscle-damaging exercise protocols such as 100 drop jumps [44] or 75 eccentric muscle contractions [45], and higher compared to that induced by a single basketball game (~400 IU) [6,46]. Although no statistically meaningful differences between groups were observed, CK demonstrated a 3- to 5-fold rise on game days and recovery at 72 h in the Mod PT group. This suggests that increasing the playing time during a congested schedule might increase the accumulation of muscle trauma and extend the time required for the cell membrane to reach homeostasis. It should be acknowledged that this observation is based on the response of CK, an indirect marker of muscle trauma. However, CK represents a hallmark of exercise-induced muscle trauma, and its rise in serum is proportionate to the severity of muscle trauma [45].

Performance deterioration is a well-defined consequence of exercise-induced muscle trauma [47]. Accordingly, lower-limb muscle function is impaired after a single basketball game and characterized by reduced muscle strength for 48 h [6]. Interestingly, Delextrat et al. [48] revealed that after a single game, isokinetic peak torque is reduced to a greater extent in knee flexors compared to knee extensors. Indeed, this observation relates to the fact that knee flexors are more vulnerable to muscle trauma because basketball includes a great number of repeated, high-intensity actions involving the stretch–shortening cycle, such as changes of direction, accelerations/decelerations, sprints, and jumps [1,2,3], during which knee flexors contract eccentrically to limit the knee’s extension and hip’s flexion during the landing phase [6]. In light of this evidence, muscle function was extensively examined in the present study, comprising both isometric and isokinetic peak torque testing of knee extensors and flexors in the dominant limb at two angular velocities (60°/s and 180°/s). Interestingly, we found that both eccentric and concentric peak torque was impaired up to 24–48 h post-G3, but the reduction was more pronounced at 60°/s compared to 180°/s. This velocity-specific deterioration in isokinetic performance can be explained by the fact that during slow-speed, high-load muscle contractions, the time under tension is longer, and there is greater recruitment of high-threshold fast-twitch motor units [49], which are more susceptible to muscle trauma [50,51]. Playing time affected only the eccentric peak torque of knee flexors at 60°/s, which markedly declined at 24 h following each game in the High PT group. As mentioned above, knee flexors are eccentrically contracted during high-intensity actions, including the stretch–shortening cycle [1,2,3], and thus increased playing time during repeated games leads to muscle function impairment in a contraction-specific manner. Regardless of playing time, we observed a prolonged decline in isometric peak torque of knee flexors (up to 72 h post-G3) compared to knee extensors (recovered at 24–48 h). This finding is rather expected, considering the observation by Delextrat et al. [48], and indicates that knee flexors require more time to fully recover following a congested schedule. No differences in strength progression were detected between the trials, potentially due to the study’s limitations, which are discussed below.

Protein supplementation failed to elicit any effect on CK and muscle function during the congested schedule. Increased protein intake has been extensively examined as a dietary strategy to facilitate exercise-induced muscle trauma, given its effectiveness in stimulating muscle protein synthesis [22,23]. According to a recent systematic review with meta-analysis, increased peri-exercise protein intake can preserve muscle strength and mitigate the rise of CK concentration in the bloodstream following resistance exercise-induced muscle trauma [23]. However, these effects are evident during recovery from a single exercise bout, whereas in the present study, repeated games were employed on consecutive days, resulting in accumulated muscle trauma due to insufficient recovery. Indeed, Draganidis et al. [4] reported that milk protein supplementation during recovery from a single eccentric exercise bout mitigated the decline in strength, while previous work in soccer revealed that increased protein intake facilitated the recovery of soccer-specific performance during a congested weekly microcycle consisting of two games with a 48 h recovery period in-between [27]. An intriguing finding of the present study was that supplementation with 80 g of protein on game days resulted in higher erythrocyte GSH levels compared to the isoenergetic placebo. As shown in Table 2, the milk protein supplement was rich in cysteine, glycine, and glutamic acids, the three precursor amino acids for glutathione synthesis, increasing the total daily consumption of these amino acids. Although increased GSH availability through N-acetylcysteine supplementation has been reported to attenuate the inflammatory response and preserve muscle strength during short-term recovery (48–72 h post-exercise) from an eccentric exercise bout [25,26], these effects were not evident in the present study. This discrepancy, however, is due to disparate exercise patterns (single eccentric exercise bout vs. a congested schedule consisting of three consecutive basketball games) rather than the supplement per se (N-acetylcysteine vs. milk protein). Furthermore, it has been found that elevated levels of GSH in the body initially counteract oxidative stress but do not allow for the complete recovery of the muscular system, possibly due to the reduced activation of redox-sensitive signaling pathways [25].

A major limitation of the present study is that external overload during the basketball games was not monitored due to a lack of the appropriate instrumentation, such as internal measurement units or a global positioning system with an indoor monitoring function. This fact does not allow us to make safe conclusions, as beyond playing time, the activity pattern and overall external load during the games might have interfered with muscle function and CK response. Another secondary limitation is that performance was not assessed using field-based tests. Instead, strength was evaluated through isokinetic testing, despite its limited relevance to basketball-specific movement patterns. This method was chosen to specifically assess the eccentric muscle action of the hamstrings, a muscle group that is significantly taxed during changes of direction and deceleration. Additionally, the study involved semi-professional players who underwent comparable performance tests; however, we cannot be certain that their physical performance during matches matched the demands of these tests. Future research should include monitoring workload during games in professional basketball players to provide more comprehensive insights.

## 5. Conclusions

This study investigated the combined effects of protein supplementation and playing time within a microcycle of consecutive basketball games. The results and discussion revealed that the cumulative load from continuous participation in basketball matches was the most significant factor influencing EIMT and performance up to three days post-games. Regarding the impact of protein on performance and EIMT, the findings remain inconclusive due to the lack of load assessment during the games. Therefore, implementing a player load management process is recommended, provided it is feasible for the coaching staff.

## Data Availability

The data used in this study are confidential and cannot be shared due to stringent primary regulations and ethical considerations. Access to the data is strictly restricted to the research team so we can protect the participants’ identity and well-being.
